# Harnessing chloroplast SSRs to decipher genetic diversity in underutilized *Allium* species

**DOI:** 10.3389/fpls.2025.1645145

**Published:** 2025-09-15

**Authors:** Yogesh P. Khade, Pawan Mainkar, Aniket Chandanshive, Krishna Madhav Rai, Shalaka R. Sinhasane, Manisha Jadhav, Amol Patil, Vivekanand L. Hembade, Auji Radhakrishna, Sanket J. More, Anil Khar, Hem Raj Bhandari, Amar Jeet Gupta, Rajiv B. Kale, Krishna Prakash, Vijay Mahajan

**Affiliations:** ^1^ Indian Council of Agricultural Research (ICAR)-Directorate of Onion and Garlic Research, Pune, Maharashtra, India; ^2^ Mahatma Phule Krishi Vidyapeeth, Rahuri, Maharashtra, India; ^3^ Indian Council of Agricultural Research (ICAR)-NBPGR Regional Station, Bhowali, Uttarakhand, India; ^4^ Indian Council of Agricultural Research (ICAR)-IARI Regional Station, Pune, Maharashtra, India; ^5^ Indian Council of Agricultural Research (ICAR)-Indian Agriculture Research Institute, Hazaribagh, Jharkhand, India

**Keywords:** cp-SSR, *Allium fistulosum*, underutilized species, population structure, cross transferability

## Abstract

Alliums, including vital crops such as onion, garlic, chives, bunching onion, and leek, are globally prized for their culinary applications and medicinal attributes. However, their genetic improvement remains constrained by large genome size, high heterozygosity, and limited characterization of genetic resources. To bridge this gap, we developed chloroplast simple sequence repeat (cp-SSR) markers, which are particularly suitable for population genetics studies because of their maternal inheritance, low recombination rates, and high variability. Leveraging the chloroplast genome of *Allium fistulosum*, we identified 22 cp-SSR loci, with tetranucleotides being the most prevalent, followed by di-, tri-, and pentanucleotides. Screening 96 underutilized *Allium* accessions using polymorphic cp-SSR markers revealed 89.2% polymorphism, indicating substantial genetic diversity. The polymorphism information content (PIC) ranged from 0.00 to 0.66 (average 0.20), confirming the utility of these markers in diversity assessments. The population structure analysis revealed three distinct genetic clusters, whereas phylogenetic analysis categorized the accessions into six major clades, mirroring their evolutionary divergence. Fixation index (F_ST_) analysis showed high genetic differentiation (mean F_ST_ = 0.6) among accessions. These findings underscore the significance of cp-SSRs in revealing genetic structure and diversity across underutilized *Allium* species. This work lays a crucial foundation for integrating chloroplast markers with nuclear genomic and omics tools to drive the development of resilient, high-value cultivars suited to future agricultural challenges.

## Introduction

The genus *Allium*, comprising approximately 750 underutilized species (Fritsch and Friesen, 2002), is the largest within the monocot group and includes numerous economically important plants. The key species include onion (*Allium cepa* L.), garlic (*Allium sativum* L.), chives (*Allium schoenoprasum* L.), leek (*Allium porrum* L.), and bunching onions (*Allium fistulosum* L.) (Khade et al., 2022). In addition to these well-known crops, the genus also encompasses lesser-known species, such as *A. altaicum*, *A. ramosum*, *A. chinense*, and *A. tuberosum* etc. which are of interest for their potential contributions to biodiversity research. In addition to their agricultural value, *Allium* species hold significant ecological importance, with some listed in the Red Book of countries such as Mongolia, Russia, and China due to concerns over their conservation status (Mandakh et al., 2020).

Underutilized *Allium* species exhibit significant chromosomal diversity, ranging from diploid to highly polyploid forms. This remarkable cytogenetic variability reflects the genus’ complex evolutionary history and dynamic genomic architecture, with origins tracing back to regions of Asia and Europe. Over time, these lesser-studied species have adapted to a wide array of ecological niches, resulting in a rich spectrum of phenotypic traits and specialized adaptations. Investigating their chromosomal profiles and evolutionary trajectories not only enhances our understanding of *Allium* genomics but also supports crop improvement efforts and biodiversity conservation. Notably, underexploited *Allium* populations represent untapped reservoirs of genetic diversity, offering valuable traits for sustainable breeding and long-term genetic resource management.

To date, the genetic diversity of onions and related *Allium* species has been examined using various molecular markers, including RAPDs (Kutty et al., 2006; dos Santos et al., 2012), ISSRs (Monteverde et al., 2015; Sudha et al., 2019; Brahimi et al., 2022, 2024), and combinations of RAPD and ISSR (Sudha et al., 2019), RAPD and PCR-RFLP (Arifin et al., 2000), and RAPD and SSR (Mohapatra et al., 2023). Additional marker systems such as RFLPs (McCallum et al., 2001), AFLPs (Van Heusden et al., 2000; Simó et al., 2014), TRAP (Kisha and Cramer, 2011; Anandhan et al., 2015), SRAP combined with ISSR (Hancı and Paşazade, 2025), ILP (Gowd et al., 2023; Jayaswall et al., 2024), and SRAP (Khade et al., 2024; Mansour et al., 2020) have also been widely applied.

More recently, SSR and SNP markers have been extensively employed to assess genetic variation in *Allium fistulosum* (Yamashita et al., 2010), *Allium mongolicum* (Hu et al., 2022), and *Allium cepa* (Mahajan et al., 2009; Khar et al., 2011; Khosa et al., 2014; Jayaswall et al., 2019; Gupta et al., 2020; Lyngkhoi et al., 2021; Chalbi et al., 2023; Habsatou et al., 2024), among others. Studies incorporating STS and SNPs (McCallum et al., 2012; Scholten et al., 2016; Duangjit et al., 2013; Villano et al., 2019; Mallor et al., 2014; Hanci and Gökçe, 2016; Rivera et al., 2016; Damon and Havey, 2014; Chand et al., 2018; Labate et al., 2020; Sahoo et al., 2023) further highlight the growing utility of these high-resolution markers in understanding genetic variation. Among these, SSR markers have emerged as a preferred tool due to their high polymorphism, co-dominant inheritance, reproducibility, and cross-species transferability (Son et al., 2012; Wang and Zhang, 2022). These characteristics make SSRs effective for evaluating genetic diversity and population structure in plant species.

In addition to nuclear markers, the chloroplast genome characterized by maternal inheritance offers a powerful system for elucidating evolutionary relationships, phylogeography, and population genetics within and across *Allium* species. As a type of SSR marker, chloroplast simple sequence repeats (cp-SSRs) are particularly advantageous due to their high mutability, conservation, variability, co-dominant inheritance, and organelle-specific transmission (Ebert and Peakall, 2009). In *Allium*, cp-SSR markers have successfully revealed genetic diversity, population differentiation, and evolutionary relationships among closely related taxa (Jayaswall et al., 2023). They have also been used to detect historical demographic events, such as bottlenecks and genetic drift, which are critical for understanding population dynamics (McCauley, 1995). Strikingly, cp-SSRs contributed to onion breeding programs by facilitating targeted genetic analyses and facilitating the development of conservation strategies (Sharma et al., 2020).

Cp-SSR markers offer a non-destructive and efficient means for detecting subtle genetic variations in the chloroplast genome (Powell et al., 1995). Only a few studies have reported the use of cp-SSR markers in *Allium* species, such as *Allium cepa* L., *Allium sativum* L., and *Allium paradoxum* (M. Bieb.) (Jayaswall et al., 2022). The present study aims to investigate chloroplast genetic divergence, heterozygosity, allelic diversity, population structure, and genetic relatedness across 96 underutilized *Allium* species using 22 cp-SSR markers. By doing so, this research seeks to provide a foundation for the strategic use of these genotypes in future breeding programs and to guide the conservation of these critical plant resources in the face of ongoing environmental challenges.

## Materials and methods

### Plant material and DNA extraction

In the present study, a total of 96 underutilized *Allium* (*A.*) accessions (**Table 1**) were randomly collected from their primary regions of distribution across India. The collected samples were planted during the regular crop growing season at an experimental field site at the Indian Council of Agricultural Research-Directorate of Onion and Garlic Research (ICAR-DOGR) in Rajgurunagar, Pune, Maharashtra, India, which is located at geographic locations (18°52’0”N, 73°54’0”E; 645 m above sea level). Young leaf tissues from ten individuals per accession (96 underutilized *Allium* species) were collected randomly for genomic DNA isolation. Total genomic DNA was isolated from these samples via the CTAB method as described by Murray and Thompson (1980). Leaf tissues were homogenized in liquid nitrogen and incubated for an hour at 65°C in 1 ml of CTAB buffer, which contained 4% polyvinylpyrrolidone (PVP), 0.5% β-mercaptoethanol, 1.4 M NaCl, 100 mM Tris-HCl, 20 mM EDTA, and 2% cetyl trimethylammonium bromide. The quantity and quality of the extracted DNA were assessed by electrophoresis on a 0.8% agarose gel, using lambda HindIII marker (Thermo Fisher Scientific) used as a reference.

**Table 1:** Description of the samples used for the characterization of chloroplast derived simple sequence repeat markers (cp-SSR).

Sl. no.	Name of the species	Code	Sl. no.	Sample_name	Code	Sl. no.	Sample_name	Code
1	*Alliun cepa* L. Var. Bhima Super	AceB.Super	33	*Allium macranthum* L. NMK 3233	AmacNMK3233	65	*Allium hookeri* L.	AhooNG3155
2	*Allium sativum* L. Var. Bhima Purple	AsaB.Purple	34	*Allium macranthum* L. NMK 3232	AmacNMK3232	66	*Allium hookeri* L.	AhooNMK3235
3	*Allium altaicum* L. EC 328485 Pall	AalEC328485P	35	*Allium macranthum* L. NMK 3229	AmacNMK3229	67	*Allium hookeri* L.	AhooNG3156
4	*Allium altaicum* L. Pall CGN 14769	AalCGN14769P	36	*Allium macranthum* L.	Amacranthum	68	*Allium hookeri* L.	A.hookeri
5	*Allium cepa* var aggregatum 3 Meitai Tilou	Aceaggr3MT	37	*Allium tuberosum* L.	AtubBKCGN15749	69	*Allium auriculatum* L.	A.auriculatum
6	*Allium cepa var* aggregatum 4 Eshing Tilou	Aceaggr4ET	38	*Allium tuberosum* L.	AtubCGN16418	70	*Allium albidum* L.	A.albidum
7	*Allium cepa* var aggregatum 5 Manipur	Aceaggr5M	39	*Allium tuberosum* L.	AtubESKCGN16412	71	*Allium oreoprasum* L.	A.oreoprasum
8	*Allium cepa* × *Allium fistulosum* Beltsville Bunching	Ace × AfisB	40	*Allium tuberosum* L.	AtubKazhakistan1587	72	*Allium ramosum* L.	A.ramosum
9	*Allium fistulosum* L. NIC 23426A	AfisNIC23426A	41	*Allium tuberosum* L.	AtubZimmu	73	*Allium fasciculatum* L.	A.fasciculatum
10	*Allium fistulosum* L. NIC 20221	AfisNIC20221	42	*Allium tuberosum* L.	AtubMKG24	74	*Allium viviparum* L.	A.viviparum
11	*Allium fistulosum* L. EC 321643.1	AfisEC321643.1	43	*Allium tuberosum* L.	AtubMKG3214	75	*Allium stracheyi* L.	A.stracheyi
12	*Allium fistulosum* L. EC 321643.2	AfisEC321643.2	44	*Allium tuberosum* L.	AtubNMK3214	76	*Allium negianum* L.	A.negianum
13	*Allium fistulosum* L. AKO-1 (China)	AfisAKO-1.China	45	*Allium tuberosum* L.	AtubIC353524	77	*Allium consanquianum* L.	A.consanguineum
14	*Allium fistulosum* L. BG Autumn Nepthane	AfiBGAN	46	*Allium tuberosum* L.	AtubNG3183	78	*Allium roylei* L.	A.roylei
15	*Allium fistulosum* L.	*A.fistulosum*	47	*Allium tuberosum* L.	AtubNMK3219	79	*Allium proliferum* L.	A.proliferum
16	*Allium fistulosum* L. Georgian	AfisGeorgien	48	*Allium tuberosum* L.	Atub.OP	80	*Allium wallichii* L.	A.wallichii
17	*Allium fistulosum* L. (China) All 647	AfisAll647.China	49	*Allium tuberosum* L.	AtubNMK3231	81	*Allium barsczewskii* L.	AbarMK95
18	*Allium macranthum* L. NMK 3240	AmacNMK3240	50	*Allium tuberosum* L.	AtubNMK3228	82	*Allium senescense* L.	AsenEC328503
19	*Allium chinense* Cholang White RAK100	AChiRAK100	51	*Allium tuberosum* L.	AtubNMK3207	83	*Allium schoenoprasum* L.	AschNMK12
20	*Allium chinense* L. NMK 3247	AChiNMK3247	52	*Allium tuberosum* L.	AtubEC607483	84	*Allium schoenoprasum* L.	Aschoenoprasum
21	*Allium chinense* L.	*A.chinense*	53	*Allium tuberosum* L.	Atuberosum	85	*Allium schoenoprasum* L.	AschhNR6NGB14774
22	*Allium macranthum* L. NMK 3216	AmacNMK3216	54	*Allium prszewalskianum* L.	AprzMMK120	86	*Allium schoenoprasum* L.	AschNRNGB597
23	*Allium macranthum* L. NMK 3242	AmacNMK3242	55	*Allium prszewalskianum* L.	AprzMMK119	87	*Allium ascalonicum* L.	AascalC353523
24	*Allium macranthum* L. NMK 3248	AmacNMK3248	56	*Allium prszewalskianum* L.	AprzMMK121	88	*Allium ascalonicum* L.	AascalC99923
25	*Allium macranthum* L. NMK 125	AmacNMK125	57	*Allium chinense* L.	AChiNMK3165	89	*Allium porrum* L.	AporlC632238
26	*Allium macranthum* L. NMK 3227	AmacNMK3227	58	*Allium chinense* L.	AchiNMK3236	90	*Allium porrum* L.	Aporl353526
27	*Allium macranthum* L. NMK 3246	AmacNMK3246	59	*Allium chinenes* L.	AchiMMK131	91	*Allium altaicum L.*	AalCGN23934P
28	*Allium macranthum* L. NMK 3245	AmacNMK3245	60	*Allium ascalonicum* L.	A.ascalonicum	92	*Allium altaicum* CGN 14769	AalCGN14769P
29	*Allium macranthum* L. NMK 3244	AmacNMK3244	61	*Allium fragrance* EC 383446	AfraEC383446	93	*Allium altaicum* L. CGN 14771	AalCGN14771
30	*Allium macranthum* L. NMK 3243	AmacNMK3243	62	*Allium angulosum* EC 328486	AangEC328486	94	*Allium fragrance* L.	AfraEC383447
31	*Allium macranthum* L. NMK 3238	AmacNMK3238	63	*Allium ampeloprasum* NMK 3211	AampNMK3211	95	*Allium fistulosum* L.	Afis.OP
32	*Allium macranthum* L. NMK 3237	AmacNMK3237	64	*Allium carolinianum* L.	AcarMMK135	96	*Allium schoenoprasum* L. NR6 NGB 147745	AschNR6NGB14775

### Chloroplast SSR marker development

The simple sequence repeats (SSR) loci within the *Allium fistulosum* chloroplast genome (Voucher No. PRJNA927338; NCBI Reference ID: NC_040222.1; Omelchenko et al., 2020) were identified using the MISA tool (http://misaweb.ipk-gatersleben.de/). The cp-SSR motif analyzed consisted of repeat units ranging from di- to hexanucleotides, meeting the minimum repeat thresholds set by MISA. Specifically, six motifs contained dinucleotide repeats, four contained trinucleotides, and three included tetra-, penta-, or hexanucleotide repeats. Mononucleotide repeats were excluded from further analysis. Both perfect and compound SSRs were detected via the MISA pipeline, with compound repeats defined as SSRs interrupted by non-repeat sequences of up to 100 bp. Primer pairs flanking the cp-SSR loci were designed using the BatchPrimer3 v1.0 online tool (https://probes.pw.usda.gov/batchprimer3; You et al., 2008). The primer design parameters included primer lengths of 22–27 nucleotides, amplicon sizes of 100 to 300 bp, melting temperature ranging from 48°C to 55°C, and GC content between 40% and 70%, with an optimal GC content of 50% (**Table 2**).

**Table 2:** Distributions of cp-SSR motifs observed in the chloroplast genome of *Allium fistulosum*.

cp-SSR ID	Primer name	SSR type	SSR	Size	Start	End	Position
Afi01	cp-SSR01	p5	(TAAAA)3	15	3763	3777	trnK-UUU
Afi02	cp-SSR02	p4	(ATAA)3	12	4295	4306	trnK- rps16
Afi03	cp-SSR03	p3	(TTA)4	12	7142	7153	psbK- psbI
Afi04	cp-SSR04	p4	(ATTT)3	12	19810	19821	rpoC1-rpoC2
Afi05	cp-SSR05	p5	(ATTGA)3	15	29023	29037	pet9- psbM
Afi06	cp-SSR06	p2	(AT)6	12	31979	31990	trnT-GGU- psbD
Afi07	cp-SSR07	p2	(TA)6	12	35101	35112	trnS-UGA- psbZ
Afi08	cp-SSR08	p4	(TTTC)3	12	42789	42800	ycf3 Intron
Afi09	cp-SSR09	p2	(TA)8	16	45568	45583	rps4- trnT-UGU
Afi10	cp-SSR10	p5	(TATAA)3	15	58198	58212	accD- psaI
Afi11	cp-SSR11	p4	(AATG)3	12	60278	60289	cemA
Afi12	cp-SSR12	p4	(TAAA)3	12	61891	61902	petA- psbJ
Afi13	cp-SSR13	p2	(TA)6	12	65755	65766	psaJ- rpl33
Afi14	cp-SSR14	p3	(TCT)4	12	69000	69011	ClpP Intron
Afi15	cp-SSR15	p4	(TAAA)3	12	69831	69842	ClpP Intron
Afi16	cp-SSR16	p4	(TTTA)3	12	72681	72692	psbN- psbH
Afi17	cp-SSR17	p4	(GGAT)3	12	74759	74770	petB- PetD
Afi18	cp-SSR18	p4	(ATTG)3	12	111468	111479	ndhF- rpl32
Afi19	cp-SSR19	p3	(AAT)4	12	115962	115973	psaC- ndhE
Afi20	cp-SSR20	p2	(TA)7	14	116305	116318	psaC- ndhE

p2, Dinucleotide; p3, Trinucleotide; p4, Tetranucleotide; p5, Pentanucleotide.

### cp-SSR marker analysis

A total of 22 cp-SSR primer pairs were selected and synthesized by Eurofins Genomics (Eurofins, India). After an initial run with the newly developed primer pairs, 20 cp-SSRs exhibiting high resolution, stability, and significant polymorphism were selected for further analysis. The cp-SSR amplification was carried out in a 20 µl reaction volume, which included 2 µl of 10X reaction buffer and 50 ng of template DNA per reaction. The PCR reaction mixture consisted of 50 ng of genomic DNA (1µl), 1.5 mM MgCl_2_, 0.2 mM of each dNTP, 0.2 µM of each primer (forward and reverse), and 5 U of Taq DNA polymerase. PCR amplification was performed using a Bio-Rad iCycler thermal cycler. The cycling conditions included an initial denaturation at 94°C for 4 minutes, followed by 35 cycles of denaturation at 94°C for 1 minute, annealing at the optimized temperature specific to each primer (as listed in **Table 3**), and extension at 72°C for 40 seconds. A final extension was carried out at 72°C for 10 minutes. The PCR products were analyzed via gel electrophoresis on a 3.2% agarose gel. Bands were visualized with a 1 kb Plus DNA ladder (Thermo Fisher Scientific) as a reference and documented using a gel documentation system.

**Table 3:** Details of 20 chloroplast SSR markers, including sequences, annealing temperatures, and allele size.

Sl no.	Primer name	Primer F/R	Primer sequence 5’ to 3’	No. of bases	Tm (°C)	Allele size range
1	cp-SSR01	cp-SSR01-F	CCAGAATTAGAGCCGTAGAGC	21	50.22	160-240
2		cp-SSR01-R	CCACGACTGATCCTGAAAGG	20		
3	cp-SSR02	cp-SSR02-F	TGGCAAACCCATAATTTGAA	20	48.2	200
4		cp-SSR02-R	TGTGCCAATCCAACACAAAT	20		
5	cp-SSR03	cp-SSR03-F	TCCTCGTTCTGACCTTCCAG	20	49.8	200
6		cp-SSR03-R	TGTTGACATAGTGCCCCAAA	20		
7	cp-SSR04	cp-SSR04-F	ATAAACCCGACTTCCCAAGG	20	52.2	80-110
8		cp-SSR04-R	GAAGCCATACAGGGGTTTTG	20		
9	cp-SSR05	cp-SSR05-F	TCAGCGCAATCATTTCATTT	20	55.1	200-220
10		cp-SSR05-R	TCGCACTTATTGCTACTGCAC	21		
11	cp-SSR06	cp-SSR06-F	TGATTTTCTTGTTAATGGACGC	22	53.4	200-220
12		cp-SSR06-R	TGCATTGCTGAAACAAAACAA	21		
13	cp-SSR07	cp-SSR07-F	TGTAGAAACCTCCCGGATTG	20	54.2	100
14		cp-SSR07-R	ATTCGGACATGGAGTCGAAG	20		
15	cp-SSR08	cp-SSR08-F	ATCGTTGCTTTGAACGATGC	20	55.1	200-210
16		cp-SSR08-R	TATTTCCGGGCATTAGAACG	20		
17	cp-SSR09	cp-SSR09-F	AAACAAAGCAAAGCGAAATCT	21	52.3	180-220
18		cp-SSR09-R	CCATTTCTACAAACGTTGAGTCAC	24		
19	cp-SSR10	cp-SSR10-F	TGGGTTGTCATACATATTCGTG	22	54.2	200-260
20		cp-SSR10-R	TGTCATAGAACGGGTACCTCAA	22		
21	cp-SSR11	cp-SSR11-F	ATCGCGTATCTCCTTCGCT	19	51.2	200
22		cp-SSR11-R	CCTATCCACGAGTCTGCCAT	20		
23	cp-SSR12	cp-SSR12-F	TGCTTTTTCTCTTGTTCACCA	21	50.6	100
24		cp-SSR12-R	CTCAATGAATGACTCCCCTCT	21		
25	cp-SSR13	cp-SSR13-F	AAATGAAATACTGGAAAGAATAATTGA	27	51.4	220- 240
26		cp-SSR13-R	ACCCTTAGCCATGAACCTCC	20		
27	cp-SSR14	cp-SSR14-F	ATTCAATATGGCGAAGGCAT	20	51.4	220
28		cp-SSR14-R	GATCCTTCATTCTGGTCGGA	20		
29	cp-SSR15	cp-SSR15-F	TCAATTCGTTTCATGTCTCCA	21	52.4	200
30		cp-SSR15-R	TGGAGTATCCAGGCTCTGCT	20		
31	cp-SSR16	cp-SSR16-F	ATGGCGACTAAGGTTGCTGT	20	55.2	180
32		cp-SSR16-R	CTCAACGGTTTGTGTAGCCA	20		
33	cp-SSR17	cp-SSR17-F	ATCCTATCGGGAAGGAACAA	20	54.3	180
34		cp-SSR17-R	GCATGGCCCAATCAATAGTT	20		
35	cp-SSR18	cp-SSR18-F	GCAAAGAAAAAGTAAGAAAAGTAAGCA	27	53.8	80 -280
36		cp-SSR18-R	TTCCGTTAGCTAAGAAAAGGAACT	24		
37	cp-SSR19	cp-SSR19-F	GCAGGCTCGTACACATTGAG	20	53.5	100
38		cp-SSR19-R	TTGGTCCCTTCTGATGAACA	20		
39	cp-SSR20	cp-SSR20-F	TCACGTTGACTAATGGATCGTC	22	54.7	120- 260
40		cp-SSR20-R	TGGGCTAGCTATTGTTTCGTC	21		

### Scoring and data analysis

To ensure the accuracy of the results, each pair of primers was used for PCR amplification and electrophoresis twice, and only the cp-SSR markers with high definition and good stability were scored. The scoring was performed on the basis of the absence (0) or presence (1) of each band for all the isolates in each primer. Genetic variation at each locus was characterized in terms of the number of alleles, and the PIC value was calculated. The binary matrix was subjected to Jaccard similarity coefficient analysis using NTSYS-pc version 2.02i (Rohlf, 1998), and the unweighted pair group method with arithmetic mean (UPGMA) clustering map based on Nei’s genetic distance was constructed using MEGA X (Kumar et al., 2018). Principal component analysis (PCA) and a cluster matrix were plotted on the basis of correlation distance and average genetic linkage via the web tool Clust-Vis (https://biit.cs.ut.ee/clustvis/) (Metsalu and Vilo, 2015). Genetic diversity parameters, including minor allele frequency (MAF), observed number of alleles (Na), observed heterozygosity (Ho), expected heterozygosity (He), and PIC, were calculated by GenAlEx 6.51 software (Peakall and Smouse, 2012). GenAlEx 6.51 was used to calculate the fixation index (F_ST_), which measures the proportional increase in homozygosity. F_ST_ values range from 0 (no differentiation) to 1 (complete differentiation) (Wright, 1984). The population genetic structure was analyzed using Bayesian clustering methods via STRUCTURE 2.3.4 software. The number of populations (K) was tested sequentially from 1-10. Each run included a burn-in phase of 50,000 steps, followed by 200,000 Markov chain Monte Carlo (MCMC) iterations, which enhanced the reliability of clustering, as suggested by Nouri et al. (2021). The optimal K value was determined via the average lnP(K) and StructureSelector (https://lmme.ac.cn/StructureSelector/), revealing a significant peak in the ΔK values for the most suitable population grouping.

## Results

### Characterization of the developed cp-SSR markers

**Figure 1:**
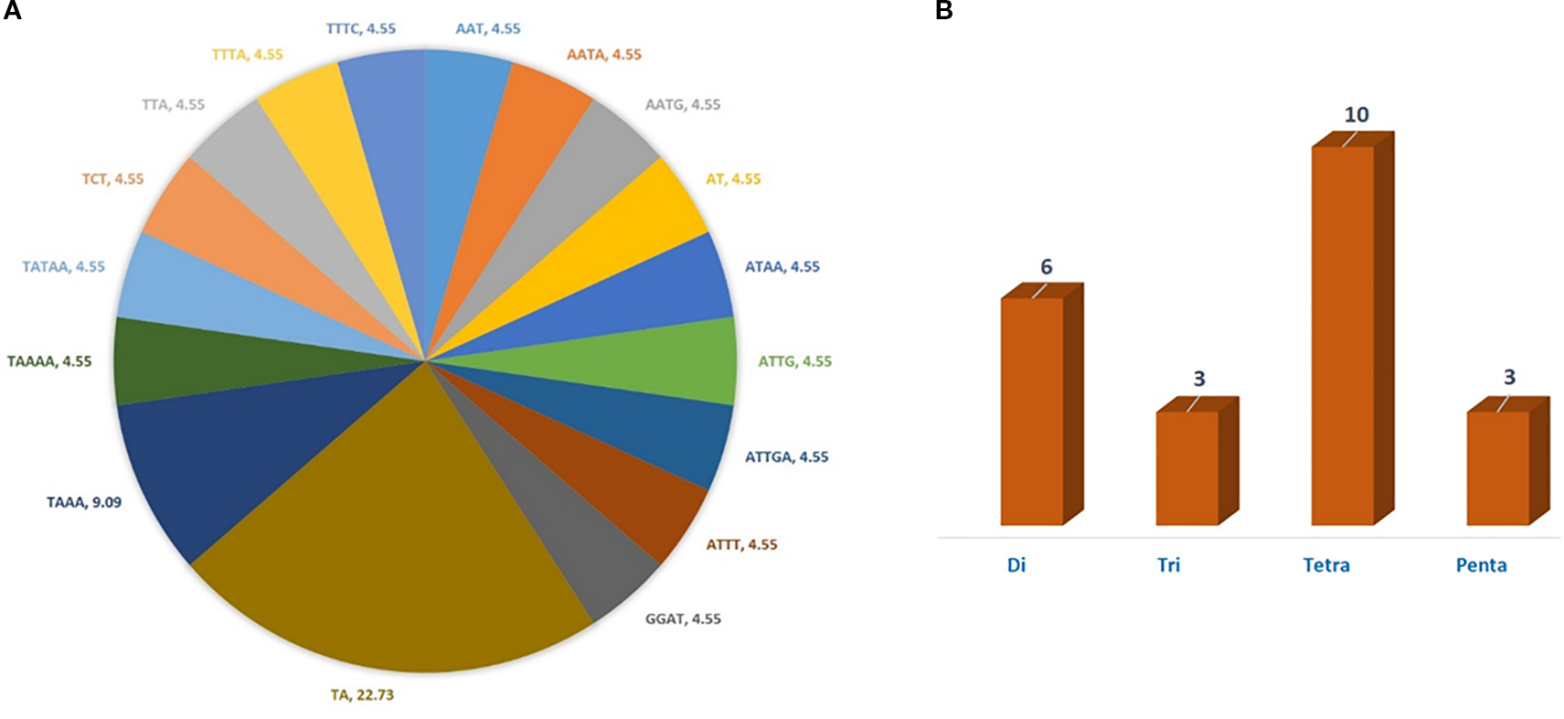
Type and proportion of motif repetition for cp-SSRs in the chloroplast genome of *Allium fistulosum*. **(A)** Distribution of different motif types in cp-SSRs. **(B)** Proportion of repeated motif types.

In the current study, a total of 22 cp-SSR marker pairs were identified from the *Allium fistulosum* chloroplast genome. Among the identified markers, tetranucleotide motifs were the most abundant (45.45%), followed by dinucleotide (27.27%), trinucleotide (13.63%), and pentanucleotide (13.63%) motifs (**Table 2** and **Figure 1**). Notably, hexanucleotide repeats were completely absent from the chloroplast genome of *A. fistulosum*. The most frequently occurring motif was TA (22.73%), followed by the TAAA motif (9.09%). All other motifs were evenly distributed. Mononucleotide repeats, primarily A/T-rich, were excluded from further analysis due to their low polymorphic potential and the risk of sequencing errors caused by homopolymer runs. The average repeat lengths for the di-, tri-, tetra-, and pentanucleotide cp-SSRs were 13, 12, 12, and 15 base pairs, respectively. Among the 22 designed primer pairs, 20 (90.91%) successfully amplified clear and reproducible bands during PCR screening with underutilized *Allium* species (**Table 3**). These markers showed high polymorphism and stability indicating their potential suitability for assessing genetic diversity in *Allium* germplasm.

### cp-SSR marker analysis

The analysis of the developed cp-SSR markers revealed a high level of polymorphism and genetic diversity among the underutilized *Allium* species (**Supplementary Figure 1**). A total of 37 allelic bands were detected using 20 polymorphic cp-SSR markers. Among these, 89.2% of the amplified alleles were polymorphic, indicating the hypervariable nature of the cp-SSR loci and the broad genetic variation in the underutilized *Alliums*. The observed allele sizes ranged from 80 to 280 bp (**Table 3**), with allele frequencies ranging from 0.00 to 0.99, demonstrating the effectiveness of these markers in capturing intra- and interspecific genetic variation. The polymorphism information content (PIC) values of the cp-SSR markers ranged from 0.00 to 0.66, with an average of 0.20 (**Table 4**). Notably, most of the markers exhibited more than 80% polymorphism, indicating their high utility for diversity and population genetic studies. These highly informative loci can serve as valuable molecular tools in future genetic analyses of *Allium* species.

**Table 4:** Statistical analyses of genetic polymorphisms in 20 cp-SSR primer pairs.

Primer name	Allele size	No. of bands	Total no. of bands present	Freq A	Freq B	PIC	Avg. PIC	%polymorphism
cp-SSR01	160	5	2	0.021	0.979	0.041	0.048	21.250
	180		1	0.010	0.990	0.021		
	200		93	0.969	0.031	0.061		
	220		5	0.052	0.948	0.099		
	240		1	0.010	0.990	0.021		
cp-SSR02	200	1	41	0.427	0.573	0.489	0.489	42.708
cp-SSR03	200	1	94	0.979	0.021	0.041	0.041	97.917
cp-SSR04	80	2	75	0.781	0.219	0.342	0.288	81.771
	110		13	0.135	0.865	0.234		
cp-SSR05	200	2	92	0.958	0.042	0.080	0.060	93.042
	220		2	0.021	0.979	0.041		
cp-SSR06	200	2	56	0.583	0.417	0.486	0.283	29.167
	220		4	0.042	0.958	0.080		
cp-SSR07	100	1	1	0.000	1.000	0.000	0.000	
cp-SSR08	200	2	89	0.927	0.073	0.135	0.088	90.042
	210		2	0.021	0.979	0.041		
cp-SSR09	180	3	11	0.115	0.885	0.203	0.191	91.389
	200		79	0.823	0.177	0.291		
	220		4	0.042	0.958	0.080		
cp-SSR10	200	4	36	0.375	0.625	0.469	0.253	62.748
	220		17	0.177	0.823	0.291		
	240		8	0.083	0.917	0.153		
	260		5	0.052	0.948	0.099		
cp-SSR11	200	1	89	0.927	0.073	0.135	0.135	92.708
cp-SSR12	100	1	84	0.875	0.125	0.219	0.219	87.500
cp-SSR13	220	2	95	0.990	0.010	0.021	0.021	95.521
	240		1	0.010	0.990	0.021		
cp-SSR14	220	1	87	0.906	0.094	0.170	0.170	90.625
cp-SSR15	200	1	9	0.094	0.906	0.170	0.170	9.375
cp-SSR16	180	1	80	0.833	0.167	0.278	0.277	83.333
cp-SSR17	180	1	70	0.729	0.271	0.395	0.395	72.917
cp-SSR18	80	3	6	0.063	0.938	0.117	0.147	13.250
	260		1	0.010	0.990	0.021		
	280		18	0.188	0.813	0.305		
cp-SSR19	160	1	0	0.000	1.000	0.000	0.000	0.000
cp-SSR20	260	3	75	0.781	0.219	0.342	0.660	95.389
	240		19	0.198	0.802	0.317		
	120		4	0.042	0.958	0.080		
	Total	37	1368				0.196	

### Data scoring and analysis

The genetic diversity analysis of 20 cp-SSR markers across 96 underutilized *Allium* species along and their respective accessions provided a comprehensive view of the genetic structure and variability within the genus. The key diversity indices assessed included the number of observed alleles (Na), effective number of alleles (Ne), Shannon’s information index (I), observed heterozygosity (Ho), expected heterozygosity (He), and unbiased expected heterozygosity (uHe) (**Table 5**). The number of alleles (Na) ranged from 1 to 4, with markers such as SSR09 and SSR10 showing the highest diversity. The effective number of alleles (Ne) ranged from 1.00 to 2.488. Shannon’s index (I) varied from 0.0 for nonpolymorphic markers to 1.1 for SSR10, indicating high intra-accession diversity for that marker.

**Table 5:** Genetic diversity analysis of cp-SSR markers across 96 wild *Allium* species.

Primer name	N	Na	Ne	I	Ho	He	uHe	F_ST_
cp-SSR01	93	3	1.067	0.157	0.065	0.063	0.063	0.0
cp-SSR02	41	1	1.000	0.000	0.000	0.000	0.000	–
cp-SSR03	94	1	1.000	0.000	0.000	0.000	0.000	–
cp-SSR04	88	2	1.337	0.419	0.000	0.252	0.253	1.0
cp-SSR05	94	2	1.043	0.103	0.000	0.042	0.042	1.0
cp-SSR06	60	2	1.123	0.222	0.017	0.110	0.111	0.8
cp-SSR08	89	2	1.023	0.062	0.022	0.022	0.022	0.0
cp-SSR09	94	4	1.606	0.757	0.000	0.377	0.379	1.0
cp-SSR10	63	4	2.488	1.097	0.048	0.598	0.603	0.9
cp-SSR 11	89	1	1.000	0.000	0.000	0.000	0.000	–
cp-SSR12	84	1	1.000	0.000	0.000	0.000	0.000	–
cp-SSR 13	95	2	1.011	0.033	0.011	0.010	0.011	-0.1
cp-SSR 14	87	1	1.000	0.000	0.000	0.000	0.000	–
cp-SSR15	9	1	1.000	0.000	0.000	0.000	0.000	–
cp-SSR16	80	1	1.000	0.000	0.000	0.000	0.000	–
cp-SSR17	70	1	1.000	0.000	0.000	0.000	0.000	–
cp-SSR18	24	3	1.707	0.700	0.042	0.414	0.423	0.9
cp-SSR20	77	2	1.308	0.398	0.221	0.236	0.237	0.1

The observed heterozygosity (Ho) was predominantly 0.000 for most of the markers, suggesting low heterozygosity levels, whereas cp-SSR10 exhibited a slightly greater value of 0.048. The expected heterozygosity (He) ranged from 0.000 to 0.598, and the unbiased expected heterozygosity (uHe) ranged from 0.000 to 0.603, both of which were highest for cp-SSR10. Markers such as cp-SSR09, cp-SSR10, and cp-SSR18 exhibited high genetic diversity, whereas cp-SSR02, cp-SSR03, cp-SSR11, and cp-SSR15 were monomorphic with no diversity (Na and Ne = 1.000; I, Ho, and He = 0.000). The fixation index (F_ST_) ranged from 0.0 to 1.0 among the accessions within the six major clades, with a mean F_ST_ value of 0.6. Cross-transferability analysis among 30 *Allium* genotypes (**Table 6**) revealed that the transferability percentage of cp-SSR alleles ranged from 55% to 90%. The highest transferability was observed in *A. viviparum* (90%), whereas *A. altaicum* Pall CGN 14769 and *A. altaicum* CGN 1477 presented the lowest transferability (55%). Other important species, such as *A. hookeri* and *A.* fragrance, presented intermediate transfer percentages of 73.75% and 72.5%, respectively.

**Table 6:** Cross-transferability observed in different underutilized *Alliums*.

Sl no.	Name of the genotype	Transferable alleles	% of transferability
1	*A. cepa* var. Bhima Super	12.00	60.00
2	*A. sativum* var. Bhima Purple	14.00	70.00
3	*A. altaicum* EC 328485 Pall	11.5	57.50
4	*A. cepa var aggr* 3 Meitai Tilou	12.00	60.00
5	*A. fistulosum*	12.45	62.27
6	*A. chinensis* NMK 3236	13.33	66.66
7	*A. macranthum* NMK 324	12.56	62.81
8	*A. tuberosum* Bawang Kuchaai CGN 15749	12.61	63.05
9	*A. prszewalskianum* MMK 12	14.00	70.00
10	*A. fragrance* EC 383446	14.50	72.50
11	*A. angulosum* EC 328486	13.66	68.33
12	*A. hookeri* NG 3155	14.75	73.75
13	*A. auriculatum*	14.00	70.00
14	*A. albidium*	16.00	80.00
15	*A. oreoprasum*	13.00	65.00
16	*A. ramosum*	15.00	75.00
17	*A. fasciculatum*	15.00	75.00
18	*A. viviparum*	18.00	90.00
19	*A. stracheyi*	16.00	80.00
20	*A. negianum*	17.00	85.00
21	*A. consanguineum*	16.00	80.00
22	*A. roylei*	15.00	75.00
23	*A. proliferum*	16.00	80.00
24	*A. barsczewskii*	13.00	65.00
25	*A. senescens* EC 3285-3	13.00	65.00
26	*A. ascalonicum* MMK 13	13.00	65.00
27	*A. porrum*	13.00	65.00
28	*A. altaicum* pall CGN 14769	11.00	55.00
29	*A. altaicum* CGN 1477	11.00	55.00
30	*A. schoenoprasum* NR 6 NGB 14774	12.00	60.00

### Genetic relationships among underutilized and cultivated *Alliums*

**Figure 2:**
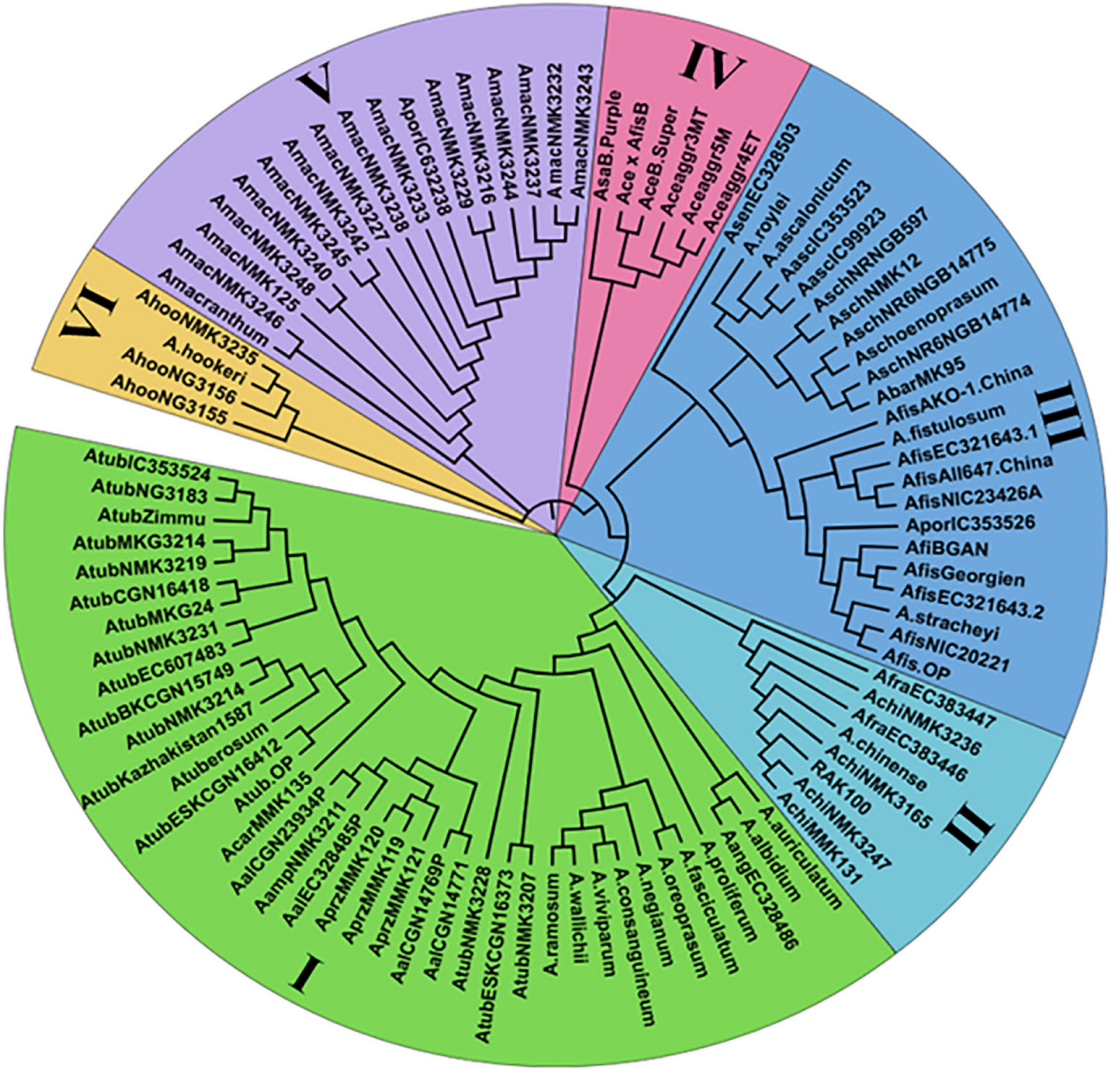
Dendrogram analysis of 96 underutilized *Allium* species based on Nei's distance of 20 novel cp-SSR markers.

Chloroplast microsatellite markers were utilized to assess genetic relationships among 96 underutilized *Allium* species through neighbor-joining (NJ) cluster analysis. The dendrogram (**Figure 2**) grouped the accessions into six distinct clusters (I–VI), each representing varying degrees of genetic relatedness. Cluster I was the largest, consisting of 38 accessions primarily representing *Allium tuberosum* and closely related taxa such as *A. ramosum*, *A. viviparum*, *A. wallichi*, *A. consanguineum*, *A. albidium*, and *A. auriculatum*. These accessions presented have high genetic similarity, likely due to shared ancestry, ecological adaptation, and geographical proximity. Cluster II included eight underutilized accessions, such as the *Allium chinense* MMK131, *Allium chinense* NMK3247, *Allium chinense* (RAK100), *Allium chinense* NMK3165, *A. chinense*, *A. chinense* NMK3236 and *A. fragrans* genotypes, which form a genetically cohesive group on the basis of habitat and cytoplasmic traits. Cluster III comprised 23 accessions dominated by *A. fistulosum*, along with related species such as *A. ascalonicum, A. stracheyi*, *A. porrum*, *A. roylei* etc. reflecting significant diversity and wide geographical origins. Cluster IV consisted of six cultivated *Allium* accessions, including landraces and hybrids (*Allium cepa* var. aggregatum 3, 4 and 5, *Allium cepa* var. Bhima Super, *Allium sativum* var. Bhima Purple, and *Allium cepa × Allium fistulosum* Beltsville Bunching, showing limited diversity due to breeding bottlenecks. Cluster V included 17 underutilized accessions, such as *A. macranthum* and its relatives, as well as *A. porrum* adapted to high-altitude environments, reflecting substantial genetic divergence. Cluster VI was the smallest, comprising four *A. hookeri* accessions showing a distinct genetic lineage. Notably, Clusters III and V presented the highest levels of intracluster genetic diversity, whereas Clusters I and IV were relatively homogeneous.

### Population structure analysis

**Figure 3:**
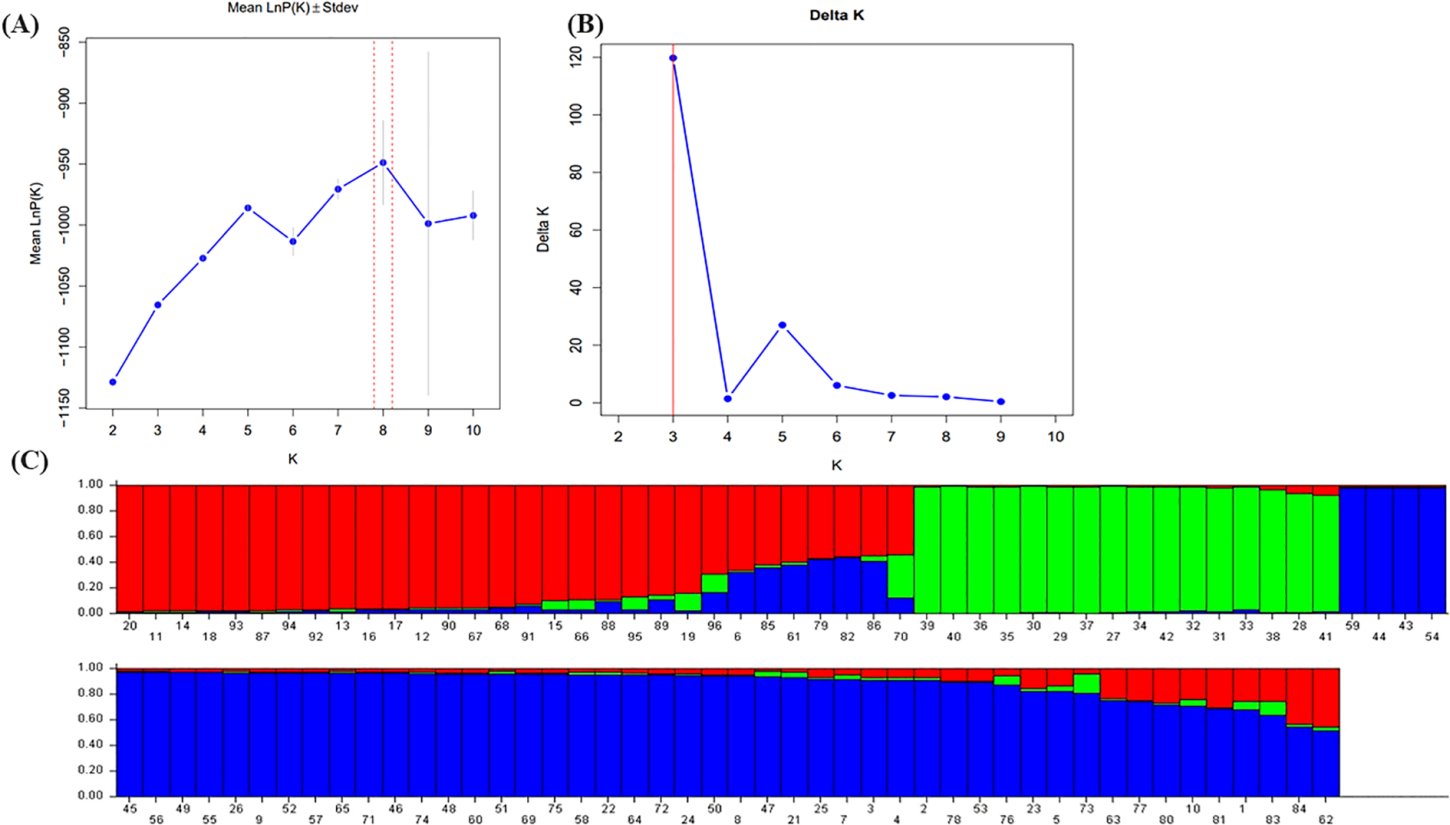
Population structure analysis of 96 wild Allium species based on 22 cp-SSR markers. **(A)** The mean value of InP **(D)** was used to estimate the population structure, and the range of *K*-values was 1-10. **(B)** Using the cuive of Δ*K* obtained by InP(K), the optimal *K*-value was determined to be 3. **(C)** The 96 underutilized Allium species studied clustered in three subgroups (subgroup I, red; subgroup II, green; and subgroup III, blue). Each histogram represents a germplasm in which different colors represent the estimated component coefficients using *Q*-values.

The population structure was analyzed using STRUCTURE v2.3.4 software based on data from 20 cp-SSR markers. The analysis was conducted for K-values ranging from 1 to 10. As K increased, the log probability of the data [lnP(K)] also increased (**Figure 3A**), and the optimal number of clusters was determined using the ΔK method (Evanno et al., 2005). A clear peak at K = 3 was observed (**Figure 3B**), indicating the most likely number of genetic clusters. Accordingly, the accessions were grouped into three distinct sub-populations: pop1, pop2, and pop3 (**Figure 3C**). The mean intracluster genetic distances for these three populations were 0.2208, 0.1664, and 0.1699, respectively, whereas the average allele-frequency divergence among populations was 0.1100. The alpha mean value was 0.051, and the proportion of membership for each cluster was estimated at 0.313, 0.185, and 0.502, respectively. These results reveal a moderate level of genetic structure and highlight substantial within-population diversity among underutilized *Allium* species.

### Principal component analysis and heatmap analysis

**Figure 4:**
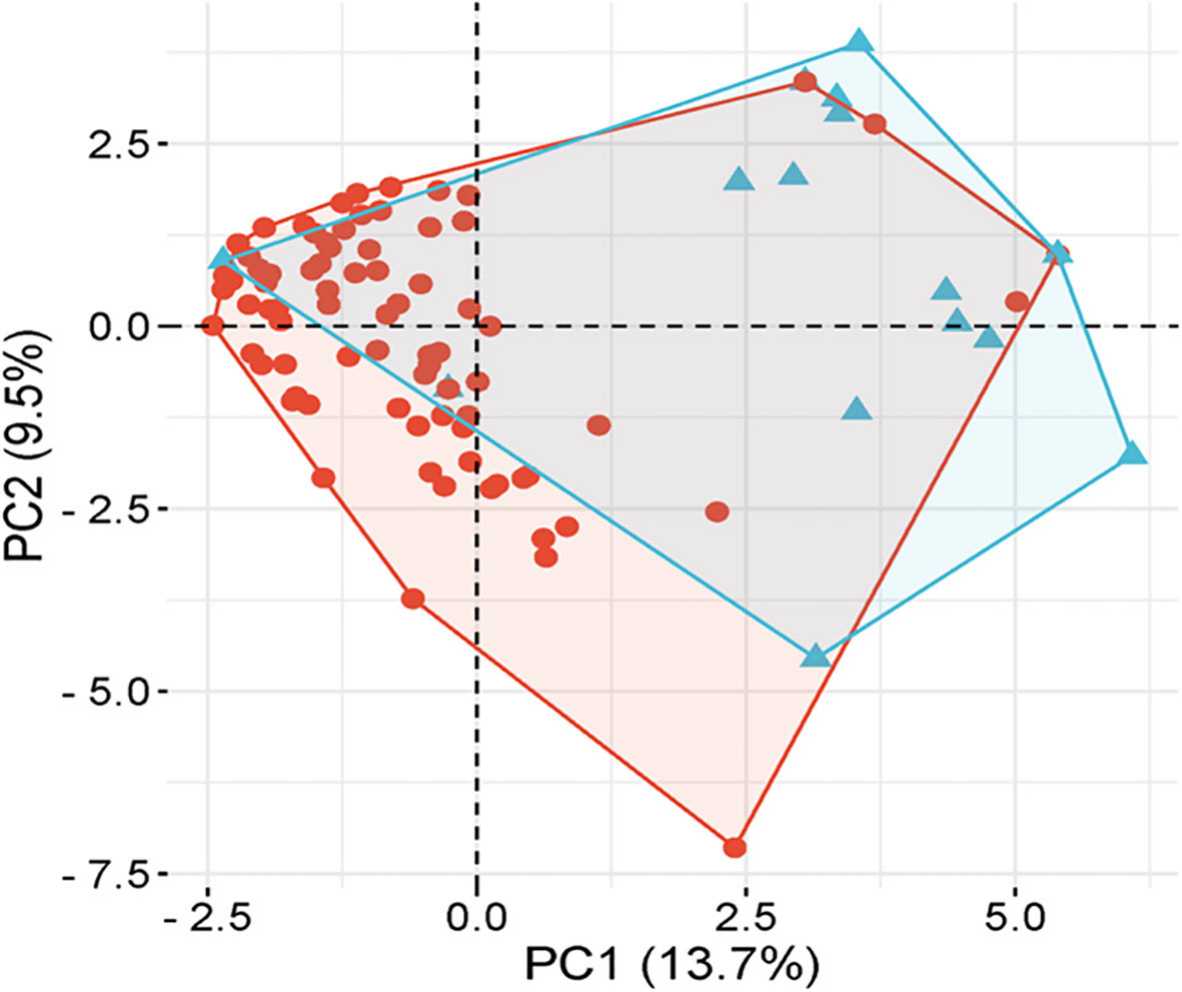
Principal component analysis (PCA) of underutilized *Allium* species.

**Figure 5:**
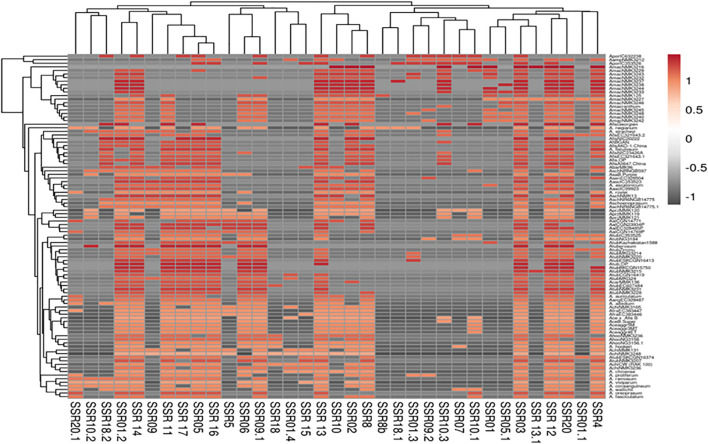
Heatmap of 96 underutilized *Allium* species based on 20 cp-SSR markers.

PCA analysis revealed that the first two principal components, PC1 and PC2, accounted for 13.7% and 9.5% of the total genetic variation, respectively (**Figure 4**). The distribution of accessions across the PCA biplot indicated the presence of two major genetic clusters. The first cluster displayed broader dispersion, primarily in the negative PC1 axis, suggesting greater genetic diversity. In contrast, the second cluster appeared more compact and tightly grouped, indicating higher genetic similarity among its members. The overlap observed between the two clusters suggested the presence of genetic admixture among certain accessions. In accordance with the PCA results, the heatmap analysis (**Figure 5**) presented a color-coded visualization of pairwise genetic similarity, with red shading indicating high similarity (values closer to 1.0) and gray shading denoting low similarity (values closer to -1.0). The accompanying hierarchical clustering dendrogram revealed distinct clusters of genetically similar accessions, whereas vertical patterns across the heatmap highlighted conserved genetic markers.

## Discussion

Chloroplast SSR markers have proven to be highly informative in studies of plant genetic diversity, phylogenetics, and population structure because of their uniparental inheritance, low recombination rates, and conserved genomic context. The central focus of this study was the development and characterization of chloroplast simple sequence repeat (cp-SSR) markers in *Allium fistulosum*, with subsequent amplification testing across 96 underutilized *Allium* species. A total of 22 novel cp-SSR loci were identified and validated, revealing distinctive patterns in repeat motif distribution that contribute to our understanding of cp-SSR evolution and utility within the *Allium* genus. Our findings revealed that tetranucleotide repeats were the most abundant (45.45%), followed by dinucleotide repeats (27.27%). Notably, mononucleotide repeats were deliberately excluded due to their lower informativeness and susceptibility to sequencing errors and polymerase slippage, a strategy aligned with best practices in cp-SSR marker development (Li et al., 2020). This exclusion also avoided overrepresentation of polyA/polyT stretches, which are highly abundant but offer limited polymorphic information. Furthermore, hexanucleotide motifs were absent, consistent with previous cp-SSR studies where longer motifs are generally rare in chloroplast genomes.

Our results partly align with prior studies in *Allium* species. For instance, Jayaswall et al. (2022) reported the development of 22 cp-SSRs in *Allium cepa* and *Allium sativum*, and 15 cp-SSRs in *Allium paradoxum*. In contrast to our current findings, their work found that tri-nucleotide repeats were the most frequent motif type (50%), suggesting potential interspecific variation in repeat motif composition within the genus *Allium*. This difference could be attributed to genomic structural variation or differing evolutionary pressures across species. When compared to broader angiosperm studies, our cp-SSR motif composition is consistent with patterns observed in other taxa. For example, Guo et al. (2022) identified 139 cp-SSR loci across 11 tree peony plastomes, while Shukla et al. (2018) reported 21–25 cp-SSRs in various *Vigna* species (*V. angularis*, *V. radiata*, and *V. unguiculata*), and Huang et al. (2018) found 92 SSR loci across six *Cupressaceae* plastomes. In these studies, di- and tetranucleotide repeats also predominated, underscoring a conserved pattern of SSR distribution in chloroplast genomes across plant lineages.

Interestingly, our findings contrast with those of Feng et al. (2023) in *Physalis angulata*, where mononucleotide repeats were the most abundant (68.24%), followed by tetranucleotides (12.28%). The abundance of mononucleotide motifs in that study likely reflects a different analytical approach that included these repeats, which, while common, are typically avoided in marker development due to their lower polymorphic potential. Overall, the distribution of cp-SSR motif types in *Allium fistulosum* reflects both conserved and species-specific patterns observed across plant taxa. Our deliberate methodological choices such as excluding mononucleotide repeats support the development of highly informative, polymorphic, and stable markers, which are essential for downstream applications such as population genetics, phylogenetic reconstruction, and genetic diversity studies in *Allium* and related genera. This work contributes to the growing genomic toolkit for *Allium* research and supports future efforts in conservation and breeding of underutilized species. The high success rate of amplification (90.91%) and clear electrophoretic profiles of these cp-SSR markers demonstrate their robustness and reliability for genetic analysis. Similar success has been reported in other species, such as tree peonies, where 19 out of 21 cp-SSR markers amplified strongly (Guo et al., 2022). The cp-SSR markers developed here complement existing nuclear SSRs by capturing maternal lineage information, thereby enhancing the resolution of genetic diversity studies in *Allium* species. Moreover, these markers are expected to support broader applications in phylogeography, conservation genetics, and breeding. As more chloroplast genome sequences become available, the cross-transferability and expansion of cp-SSR marker sets will continue to facilitate species-specific and cross-species analyses (Huo et al., 2019; Feng et al., 2023). This study lays the groundwork for future genomic research and supports the strategic use of cp-SSR markers in *Allium* crop improvement and biodiversity assessment programs.

The cp-SSR marker analysis confirmed the effectiveness of the developed markers in revealing polymorphisms and genetic variation within *Allium* germplasm. The high rate of polymorphism (89.2%) and broad allele size range reflect the utility of these markers for studying genetic structure and relationships, especially in underutilized populations. The average PIC value (0.20) aligns with previous findings by Jayaswall et al. (2023), who reported PIC values ranging from 0.007 to 0.427 in *Allium* germplasm via chloroplast-derived SSR markers. Although slightly lower than in studies by Gowd et al. (2024) (PIC: 0.24–0.98; avg. 0.608), Mallor et al. (2014) (avg. 0.64), and Hanci and Gökçe (2016) (up to 0.7), the moderate PIC values in this study may be attributed to differences in genome source (chloroplast vs. nuclear SSRs), marker selection criteria, and the genetic backgrounds of the tested accessions.

Similar studies have reported varying PIC values, with Baldwin et al. (2012) and Rivera et al. (2016) reporting averages of 0.45 and 0.51, respectively. Lyngkhoi et al. (2021) reported 53 alleles using 145 SSR markers, with PIC values ranging from 0.219 to 0.715 and an average of 3.54 alleles per locus. Khar et al. (2011) reported PIC values ranging from 0.00 to 0.89 with 60 primers, detecting 54 alleles across 19 primers, with an average of 2.84 alleles per locus. These comparisons highlight the impact of population structure, genomic origin, and SSR motif type on marker informativeness. Highly polymorphic markers such as cp-SSR3 and cp-SSR14, with more than 80% polymorphism, offer strong potential for use in genetic mapping and diversity studies. Similar findings by Gowd et al. (2024), where 92 polymorphic loci were identified using 19 SSR markers across 95 *Allium* accessions, underscore the importance of SSRs in understanding genetic variation.

Jayaswall et al. (2023) further demonstrated the utility of chloroplast-derived SSR markers in *A. cepa* and *A. sativum*, reporting heterozygosity values ranging from 0.009 to 0.540 and PIC values ranging from 0.007 to 0.427. These markers offer a reliable platform for evaluating genetic relationships between underutilized and cultivated *Allium* species. The observed genetic diversity in underutilized *Allium* accessions holds critical value for crop improvement. Traits such as disease resistance, yield enhancement, and abiotic stress tolerance can be introgressed from underutilized relatives into cultivated backgrounds. Therefore, the conservation and characterization of underutilized *Allium* germplasm remains essential for the resilience and sustainability of breeding programs. Recent advances underscore the complementary role of cp-SSRs markers alongside genomic tools in exploring *Allium* genetic diversity and evolutionary history (Khosa et al., 2015; Jayaswall et al., 2019; Gowd et al., 2024). The integration of cp-SSR data with nuclear SSR and genome-wide SNP dataset will further enrich our understanding of the genetic makeup of *Allium* species and support targeted breeding and conservation strategies. The observed number of alleles (Na) and effective number of alleles (Ne) support the existence of moderate polymorphism across the cp-SSR markers used. Markers such as cp-SSR09 and cp-SSR10, which exhibited relatively higher number of alleles and diversity indices, are particularly useful in revealing genetic differences among *Allium* accessions. In contrast, monomorphic markers such as cp-SSR02, cp-SSR03, and cp-SSR11 are likely associated with conserved regions of the chloroplast genome, offering limited intraspecies resolution but potential value for interspecific or phylogenetic studies (Gowd et al., 2024; Karic et al., 2018).

Generally, low Ho values align with the uniparental (mostly maternal) inheritance and haploid nature of the chloroplast genome, as well as the self-pollinating behaviour of many *Allium* species (Lyngkhoi et al., 2021). Nevertheless, the high He and I values of cp-SSR10 demonstrate its potential for distinguishing diverse genotypes and tracking lineage relationships. These findings are consistent with previous cp-SSR studies in *Allium*, where allele numbers typically ranged between 2 and 5 (Lyngkhoi et al., 2021), and in other genera, such as *Ziziphus* (Huang et al., 2015). Compared with nuclear SSRs and EST-SSRs, cp-SSRs tend to be less polymorphic, because they are located in more conserved regions of the genome (Ricciardi et al., 2020; Baldwin et al., 2012; Khar et al., 2011). However, their high cross-species transferability and evolutionary stability make them ideal for phylogenetic studies and for characterizing maternal lineages.

The cross-transferability results indicate a broad genetic base within the genus *Allium*. High transferability rates in species such as *A. viviparum* and *A. negianum* suggest their close genetic relationships with other members of the genus and their potential utility in breeding programs. In contrast, lower transferability in accessions such as *A. altaicum* indicates possible genomic divergence or evolutionary distance. These patterns of allele sharing and divergence can be exploited for both germplasm conservation and introgression breeding strategies aimed at enhancing stress tolerance or other desirable traits. Overall, the cp-SSR markers were effective in evaluating genetic diversity, understanding evolutionary relationships, and identifying candidate accessions for conservation and breeding. The combination of highly polymorphic and conserved markers allows a dual-purpose application: detailed intraspecies diversity analysis and broader phylogenetic studies across underutilized and cultivated *Allium* species.

Owing to their uniparental inheritance and lack of recombination, chloroplast SSR markers are well-suited for studying genetic relationships, evolutionary history, and domestication processes in plants (Sharma et al., 2020a). In the present study, these markers effectively distinguished underutilized and cultivated *Allium* species into six well-defined genetic clusters. The genetic homogeneity of Cluster I highlights the close relatedness among *A. tuberosum* and its allies, likely due to shared ancestry and cultivation across similar ecological regions, which aligns with the findings of Jayaswall et al. (2023), who reported similar clustering patterns. Cluster II, composed of various underutilized accessions, including *A. chinense* and *A. fragrans*, reflects cytoplasmic similarity and ecological coherence, corroborating earlier findings by Gowd et al. (2024). The presence of both cultivated and underutilized species in Cluster III emphasizes its potential as a genetic bridge, with *A. fistulosum* and related species offering valuable traits such as disease resistance and abiotic stress tolerance. Cluster IV, comprising cultivated *A. cepa* genotypes, exhibited reduced diversity, a consequence of domestication and selective breeding, which is consistent with domestication bottlenecks observed by Jayaswall et al. (2023). Cluster V presented the greatest genetic divergence, harboring underutilized species adapted to niche environments such as high altitudes, in agreement with findings from Nanda et al. (2016) and Gowd et al. (2024), highlighting their value as reservoirs of unique alleles. Cluster VI, comprising *A. hookeri*, presented species-specific genetic uniformity, likely due to restricted geographical distribution and domestication. Despite their narrow diversity, the members of this cluster possess unique traits that are valuable for region-specific applications. The high genetic variation observed in Clusters III and V underscores the evolutionary potential of underutilized species, reaffirming the importance of integrating underutilized relatives into breeding programs to increase stress tolerance, disease resistance, and adaptability in cultivated *Allium* species.

Understanding population structure is critical for effective germplasm conservation, trait mapping, and breeding applications. The identification of three distinct genetic clusters among the 96 underutilized *Allium* accessions aligns with earlier findings by Jayaswall et al. (2023), who also reported three subpopulations using cp-SSR markers in *Allium* accessions. The moderate allele–frequency divergence (0.11) and varying intracluster distances observed in this study suggest both shared ancestry and independent evolutionary trajectories among the populations. The relatively high proportion of membership in pop3 (50.2%) suggests a broad and genetically diverse group, potentially encompassing accessions with mixed ancestry. In contrast, pop2, with a lower proportion (18.5%), may represent a more genetically uniform or isolated subset. The findings also correlate with those of Lyngkhoi et al. (2021), who identified two groups in 96 underutilized *Allium* accessions through STRUCTURE analysis and five groups via discriminant analysis, illustrating how methodology and marker type influence the resolution of population structure. Gowd et al. (2024) reported four clusters using nuclear SSRs, highlighting differences attributable to marker origin (chloroplast vs. nuclear). Similarly, Chalbi et al. (2023) reported population differentiation in *Allium* landraces based on accession type rather than phenotypic traits. These collective observations underscore the utility of cp-SSR markers in deciphering maternal lineage and cytoplasmic diversity, which are particularly important for breeding strategies involving cytoplasmic male sterility or other organelle-linked traits. Thus, marker-based population structure analysis not only facilitates an understanding of genetic diversity but also provides a valuable framework for selecting parental lines and managing *Allium* germplasm effectively.

Together, the PCA and heatmap analyses provided a nuanced understanding of the genetic diversity and structure within the underutilized *Allium* accessions. The separation along PC1 likely reflects deep evolutionary divergence, whereas PC2 captures more recent or subtle genetic differentiation. The broader dispersion observed in one cluster indicates high intragroup variability, potentially representing genetically diverse underutilized relatives with adaptive significance. Moreover, the tighter grouping of the second cluster suggests a subset of accessions with conserved genomic features, possibly shaped by shared ancestry or ecological adaptation. These observations are consistent with common patterns in plant population genetics, where variable levels of diversity are often observed within and among clusters (Spanoghe et al., 2020). Notably, the PCA-based clustering results corresponded well with the three subpopulations identified through STRUCTURE analysis, supporting the robustness and complementary nature of both methods. The overlapping zones in the PCA further corroborate previous studies reporting gene flow and admixture among *Allium* species (Xiong et al., 2022). The heatmap visualization further reinforced the PCA outcomes by graphically representing the levels of genetic similarity and divergence, with the dendrogram effectively grouping genetically close accessions. Similar integrative approaches have proven valuable in deciphering population structure and evolutionary relationships in *Allium* and other crop species (Anwar et al., 2017; Saina et al., 2023). Overall, these findings not only validate the genetic groupings but also emphasize the utility of multivariate and hierarchical clustering tools in germplasm characterization, aiding in the selection of genetically diverse and elite accessions for breeding and conservation programs.

## Conclusion

In conclusion, this study has significantly advanced our understanding of the genetic diversity of underutilized *Allium* species through the development and application of novel chloroplast SSR markers. The identification of 22 cp-SSR motifs from the *A. fistulosum* chloroplast genome, with 20 markers exhibiting high polymorphism and stability, provides a robust toolkit for genetic analysis in *Allium*. The high level of polymorphism (89.2%) observed across 96 underutilized *Allium* species underscores the effectiveness of these markers in capturing genetic variation. The population structure analysis revealed three distinct genetic clusters, complemented by phylogenetic grouping into six major clusters, which offers valuable insights into the evolutionary relationships and genetic differentiation within the genus. These findings have important implications for *Allium* conservation strategies and breeding programs, highlighting the potential of underutilized germplasm as a reservoir of genetic diversity for crop improvement. This study demonstrates the utility of cp-SSR markers in revealing the complex genetic tapestry of *Allium* species, paving the way for precision-guided conservation efforts and the development of improved cultivars with enhanced traits such as disease resistance and stress tolerance.

## Data Availability

The original contributions presented in the study are included in the article/**Supplementary Material**. Further inquiries can be directed to the corresponding authors.
