# Supplementary figures and images for: Harnessing chloroplast SSRs to decipher genetic diversity in underutilized Allium species
Source: Front Plant Sci. 2025 Sep 15;16:1645145. doi: 10.3389/fpls.2025.1645145 (PMC12477137; doi:10.3389/fpls.2025.1645145)

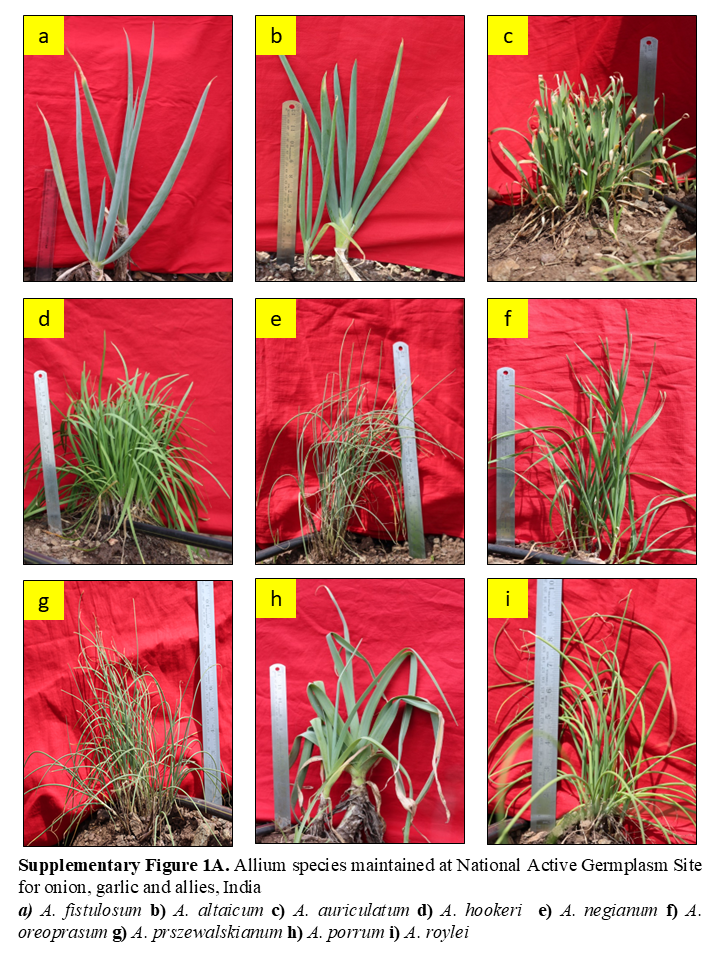

Supplement: Supplementary file 1 [file Image1.tif]

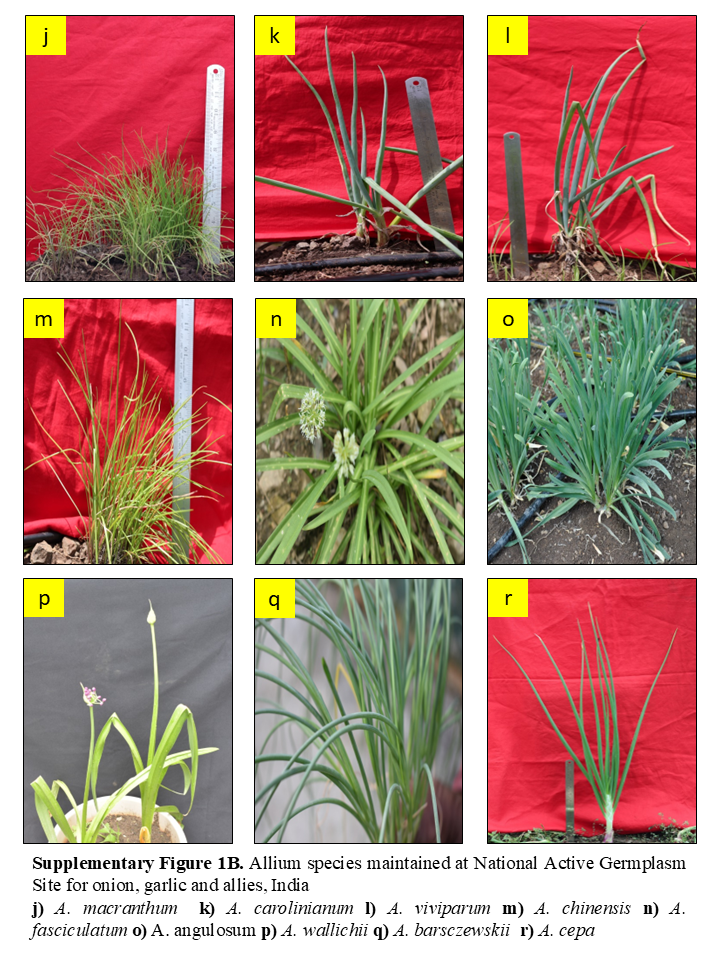

Supplement: Supplementary file 2 [file Image2.tif]

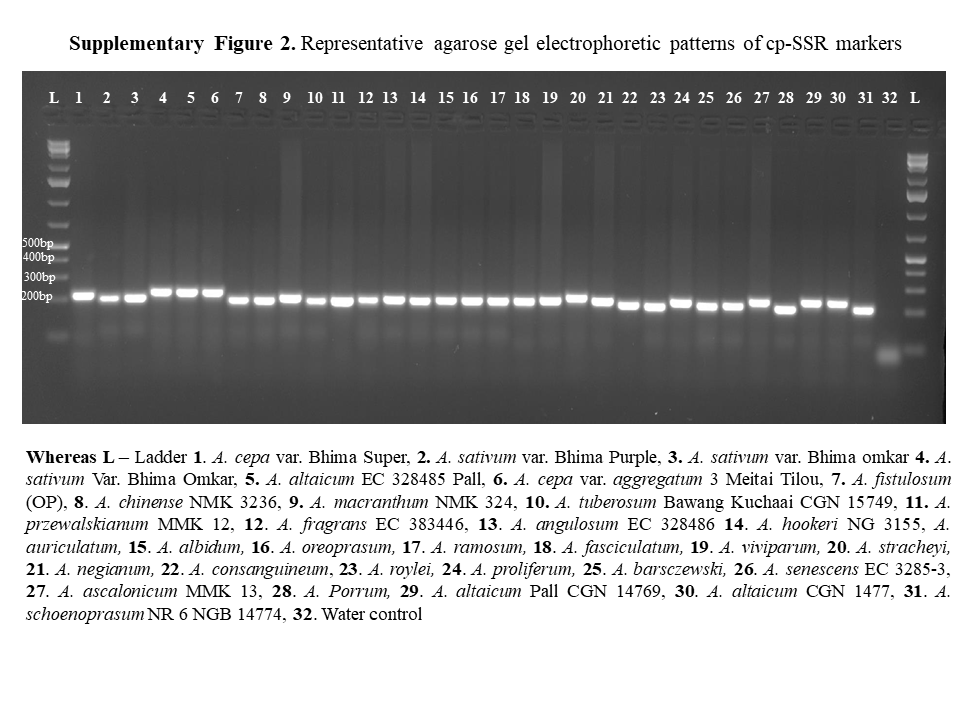

Supplement: Supplementary file 3 [file Image3.tif]
